# C-reactive protein exerts angiogenic effects on vascular endothelial cells and modulates associated signalling pathways and gene expression

**DOI:** 10.1186/1471-2121-9-47

**Published:** 2008-09-02

**Authors:** Marta M Turu, Mark Slevin, Sabine Matou, David West, Cristina Rodríguez, Ana Luque, Marta Grau-Olivares, Lina Badimon, Jose Martinez-Gonzalez, Jerzy Krupinski

**Affiliations:** 1 Department of Neurology, University Hospital of Bellvitge (HUB), Fundació IDIBELL, Barcelona, Spain; 2 Centro de Investigación Cardiovascular, CSIC-ICCC, Hospital de la Santa Creu i Sant Pau, Barcelona, Spain; 3 School of Biology, Chemistry and Health Science, Manchester Metropolitan University, Manchester, UK; 4 University of Liverpool, Liverpool, L69 7ZB, UK; 5 Hospital Sagrat Cor, Barcelona, Spain

## Abstract

**Background:**

Formation of haemorrhagic neovessels in the intima of developing atherosclerotic plaques is thought to significantly contribute to plaque instability resulting in thrombosis. C-reactive protein (CRP) is an acute phase reactant whose expression in the vascular wall, in particular, in reactive plaque regions, and circulating levels increase in patients at high risk of cardiovascular events. Although CRP is known to induce a pro-inflammatory phenotype in endothelial cells (EC) a direct role on modulation of angiogenesis has not been established.

**Results:**

Here, we show that CRP is a powerful inducer of angiogenesis in bovine aortic EC (BAEC) and human coronary artery EC (HCAEC). CRP, at concentrations corresponding to moderate/high risk (1–5 μg/ml), induced a significant increase in proliferation, migration and tube-like structure formation *in vitro *and stimulated blood vessel formation in the chick chorioallantoic membrane assay (CAM). CRP treated with detoxi-gel columns retained such effects. Western blotting showed that CRP increased activation of early response kinase-1/2 (ERK1/2), a key protein involved in EC mitogenesis. Furthermore, using TaqMan Low-density Arrays we identified key pro-angiogenic genes induced by CRP among them were vascular endothelial cell growth factor receptor-2 (VEGFR2/KDR), platelet-derived growth factor (PDGF-BB), notch family transcription factors (Notch1 and Notch3), cysteine-rich angiogenic inducer 61 (CYR61/CCN1) and inhibitor of DNA binding/differentiation-1 (ID1).

**Conclusion:**

This data suggests a role for CRP in direct stimulation of angiogenesis and therefore may be a mediator of neovessel formation in the intima of vulnerable plaques.

## Background

Atherosclerosis is the underlying cause of ischemic cardiovascular and cerebrovascular diseases [1-3]. Unstable carotid atherosclerotic plaques can undergo thrombotic complications and trigger acute clinical events [4-6]. In atherosclerotic plaques angiogenesis allows the formation of new microvessels to maintain oxygen and nutrient supply for vascular cells. Such processes are potenciated by different molecules secreted by vascular and inflammatory cells [5]. Neovessel growth occurs in active regions of atherosclerotic lesions undergoing remodelling. Our previous studies have demonstrated specific molecular deregulation occurring in these regions, consistent with the promotion of angiogenesis. The new vessels of atherosclerotic lesions may be a focus of instability, since they facilitate the infiltration of inflammatory cells and due to their tendency to leak, may produce haemorrhagic complications [5,7-9]. In a previous study using protein microarrays, we have identified regulation of potentially proangiogenic proteins associated with haemorrhagic vessels in unstable plaques among them c-Jun N-terminal kinase (JNK) and c-src [10].

The role of CRP modulating of angiogenesis in developing atherosclerotic lesions has not been investigated. CRP is an acute-phase reactant expressed during active inflammation [11-16]. Inflammation, the key regulator of CRP synthesis, plays an important role in atherothrombotic cardiovascular and cerebrovascular disease [2]. CRP consists of five identical non-covalently associated and nonglycosylated 23-kDa subunits arranged symmetrically around a central pore and it is synthesized mainly in the liver under the control of interleukin-6 (IL-6) [17,18], although it is also found in other tissues including carotid atherosclerotic plaques [5,19,20]. CRP represents one of the strongest independent predictors of symptomatic atherothrombosis and vascular death [21-23] and predicts progression of atherosclerosis [24]. In response to acute-phase stimuli, plasma CRP concentration can increase rapidly and dramatically up to 100-fold [25]. CRP levels are increased in patients at moderate- (1 to 3 μg/ml) and high-risk (> 3 μg/ml) of future cardiovascular events [21].

Although, originally CRP was suggested to be purely a biomarker, recent studies have pointed that it may in fact be a direct mediator of atherosclerosis [26]. Indeed, CRP elicits multiple effects on the vascular wall favouring a proatherosclerotic phenotype. These include attraction of monocytes, mediation of low-density lipoproteins (LDL) uptake by macrophages [27,28] reduction in nitric oxide (NO) release of human EC [29,30], up-regulation of adhesion molecules [31], stimulation of vascular smooth muscle cell (VSMC) proliferation and migration [32], increasing matrix metalloproteinase (MMP) expression in EC and macrophages [33-35], activating the complement system [36] and inducing plasminogen activator inhibitor-1 (PAI-1) expression and activity in human aortic EC [37]. With regard to CRP effects on EC, published studies are somewhat controversial. CRP has been shown to promote production of the pro-angiogenic molecules such as endothelin-1 (ET-1) and IL-6 in human saphenous vein EC [31], and activate NF-κB signalling through the CD32 receptor [38]. In contrast, other studies suggested that EC activation by CRP was due to contamination of the commercially obtained protein with lipopolysaccharide (LPS) and/or sodium azide [39,40].

As we have previously demonstrated increased CRP expression in unstable highly vascularized regions of carotid lesions, we decided to investigate the angiogenic properties of this molecule. Here we show that native CRP and not potentially contaminating LPS or azide is strongly angiogenic both *in vitro *and *in vivo*, and activates the expression of some important pro-angiogenic genes in endothelial cells.

## Results

### Chemotaxis was induced by CRP in both HCAEC and BAEC

**Figure 1:**
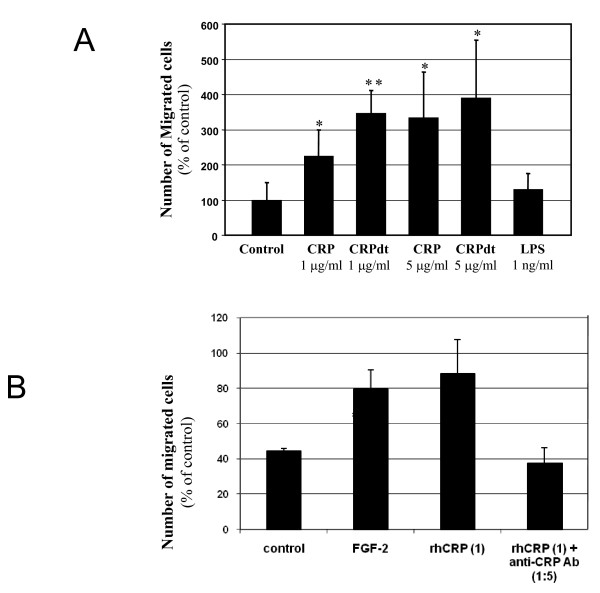
**A**, CRP induced a significant increase in chemotaxis in HCAEC. Cell migration was measured using a modified Boyden chamber as described in Methods. The effect of 1–5 μg/ml CRP (non-detoxified and detoxified CRP [CRPdt]) on HCAEC chemotaxis is shown. Endotoxin alone (LPS; 1 ng/ml) had no effect. **B**, Migration experiments were performed at least twice in triplicate wells. B, rhCRP (1 μg/ml) induced migration as compared with control and rhCRP (1 μg/ml)+antiCRP Ab(1:5). * and ** indicate a statistically significant difference compared with the control cells (*p *> 0.05 and 0.001 respectively).

Both native and CRPdt significantly increased chemotaxis of HCAEC with a maximum effect at 5 μg/ml following CRPdt treatment (approximately 290% over controls; *p *< 0.01) (Figure 1A). CRP also induced a significant increase in chemotaxis of BAEC at similar concentrations with a maximum response at 5 μg/ml CRP (approximately 175% over controls; *p *< 0.001, data not shown). FGF-2 (25 ng/ml) was used as a positive control. LPS alone (1 ng/ml) had no effect on cell migration. Blocking experiments were performed with the anti-CRP antibody (1:5) to ensure that the effect was due to the CRP protein itself. The increase in chemotaxis induced by CRPdt was significantly reduced following the pre-incubation with antibody. (Figure 1B)

### CRP stimulated BAEC but not HCAEC proliferation

**Figure 2:**
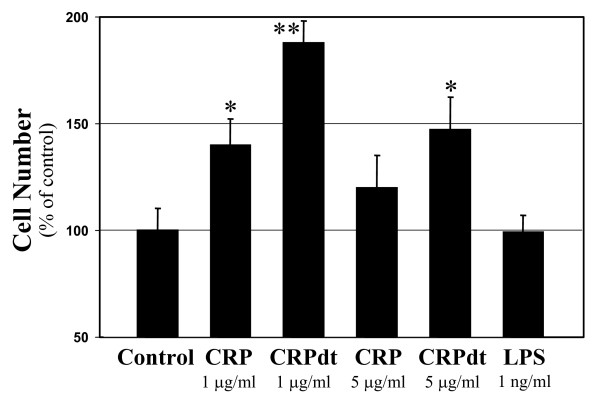
CRP stimulated BAEC proliferation. The effect of 1–5 μg/ml CRP (non-detoxified and detoxified CRP [CRPdt]) for 72 h on BAEC proliferation is shown. Experiments were repeated at least twice in triplicate wells. * and ** indicate a statistically significant difference compared with the control cells (*p *> 0.05 and 0.001 respectively).

A significant increase in BAEC proliferation (approximately 40% over controls; *p *< 0.05) was seen 72 h after treatment with 1 μg/ml native CRP (Figure 2). Treatment of CRP with detoxi-gel columns (CRPdt) did not ameliorate proliferation, and in fact, a further increase was shown (approximately 80% over controls; *p *< 0.01). At 5 μg/ml CRPdt also produced a significant effect. Endotoxin (LPS 1 ng/ml) alone had no effect on BAEC proliferation. CRP did not affect proliferation of HCAEC at any of the concentrations tested (data not shown).

### CRP induced tube-like structure formation in HCAEC and BAEC

**Figure 3:**
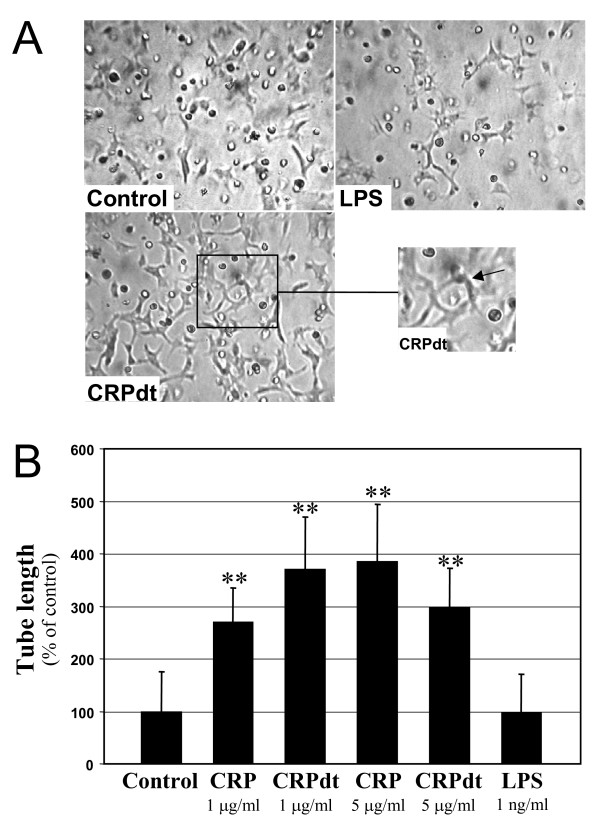
CRP induced tube-like structure formation in HCAEC. A, Representative images from control cells and cells treated with 1 ng/ml LPS or 1 μg/ml of detoxified CRP [CRPdt] for 24 h are shown. B, Bar graph showing tube length as a percentage of control. Experiments were performed at least twice by triplicate. ** indicates a statistically significant difference compared with the control (*p *< 0.001).

**Figure 4:**
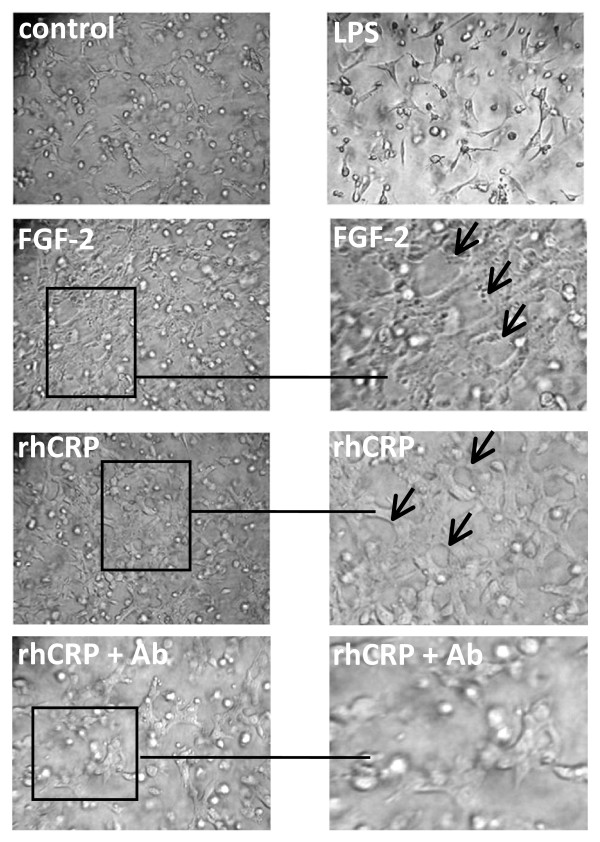
CRP induced tube-like structure formation in BAEC. Representative images from control cells and cells treated with 1 ng/ml LPS, FGF-2 (25 ng/ml), 1 μg/ml of detoxified CRP [CRPdt] or CRP + blocking antibody against CRP for 24 h are shown.

**Figure 5:**
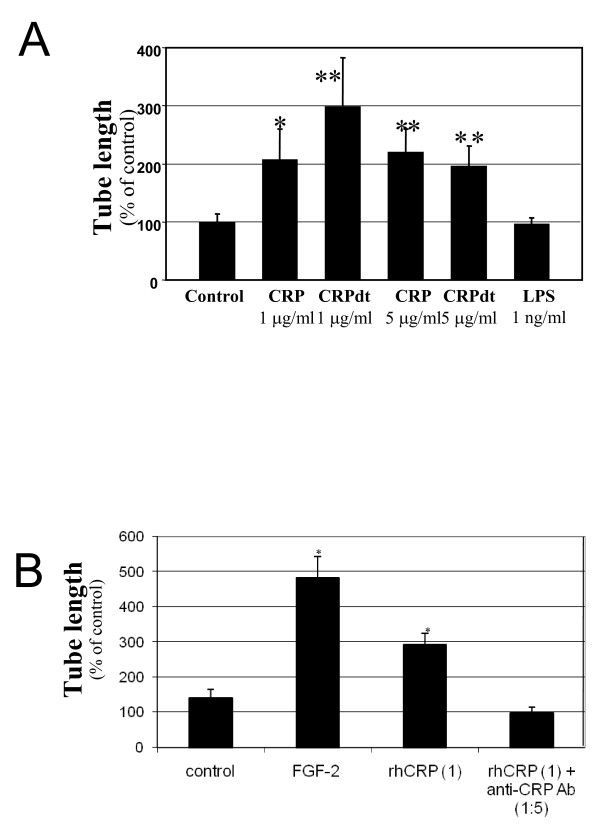
CRP induced tube-like structure formation in BAEC. A, Bar graph showing tube length as a percentage of control. Experiments were performed at least twice by triplicate. B, Bar graphs showing tube length as a percentage of control from cells treated with FGF-2 (25 ng/ml), 1 μg/ml of detoxified CRP [CRPdt] or CRP + blocking antibody against CRP for 24 h. * and ** indicate a statistically significant difference compared with the control cells (*p *> 0.05 and 0.001 respectively.

CRP induced the formation of tube-like structures in growth factor depleted matrigel in both HCAEC and BAEC. Tube formation increased in CRP-treated HCAEC by approximately 4 fold at 1–5 μg/ml (*p *< 0.01). (Figure 3). CRPdt and non-detoxified CRP produced a similar effect. LPS (1 ng/ml) had no effect. Similar results were obtained in BAEC, in this case the maximal effect was observed at 1 and 5 μg/ml with CRPdt (approximately a 3-fold increase; *p *≤ 0.01) which are lower than showed by FGF-2 treated cells (positive control)(Figure 4 and Figure 5A). Tube formation was significantly inhibited in cells pre-incubated with anti-CRP antibody, p < 0.01 (Figure 5B).

### CRP stimulated blood vessel formation in the CAM assay

**Figure 6:**
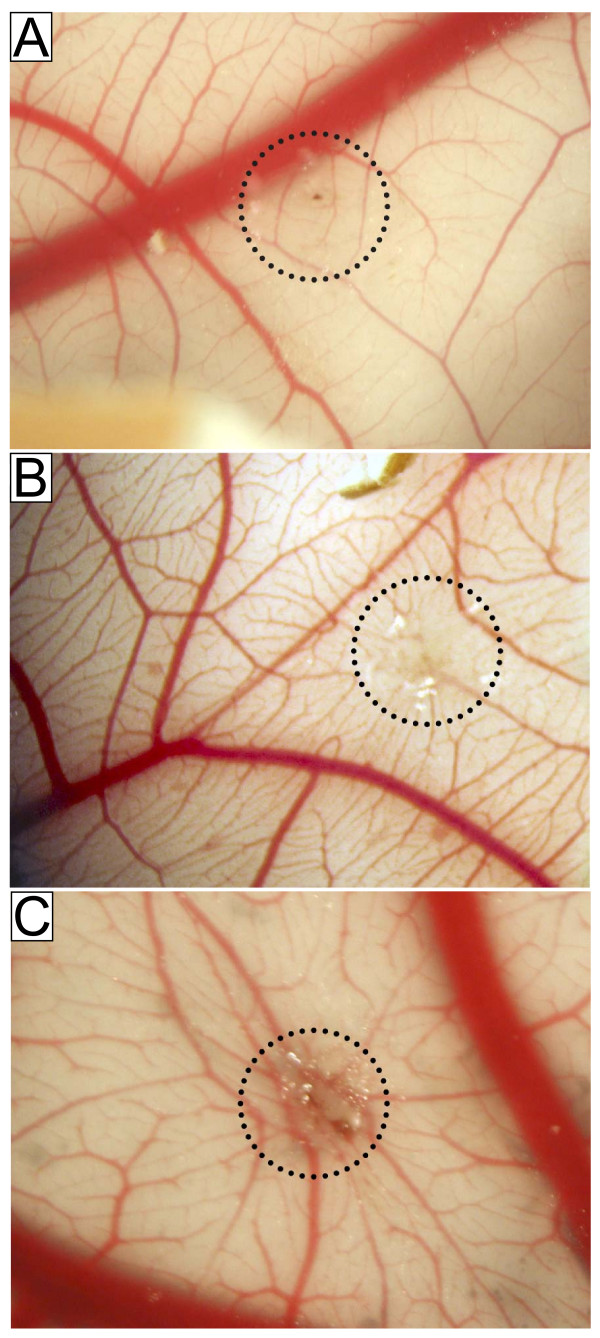
CRP stimulated spoke wheel blood vessel formation in the CAM assay. A, Negative control (score 0.5); B, CRPdt-treated (5 μg/ml; score 2.0); C, Positive control, FGF-2-treated (100 ng/ml) (score 3.0). Spoke wheel patterns can be seen in both CRP- and FGF-2-treated samples originating from the pellet (designated by dotted circles). Experiments were performed twice. A representative example of 5 CAM's used per experiment is shown.

CRPdt (5 μg/ml) was chosen following our *in vitro *studies for analysis of angiogenesis in the CAM assay. CRPdt induced blood vessel formation after 7 days treatment compared with the control, as evidenced by the spoke wheel pattern formation (mean value of 2, from 15 replicates, p < 0.001; Figure 6A, control and B, CRPdt). FGF-2 (25 ng/ml) was used as a positive control and gave a strong positive response (Figure 6C). Membranes treated with the equivalent concentration of sodium azide or with LPS (1 ng/ml) alone showed no significant response (data not shown).

### CRP stimulated angiogenic sprouting of aortic tissue

**Figure 7:**
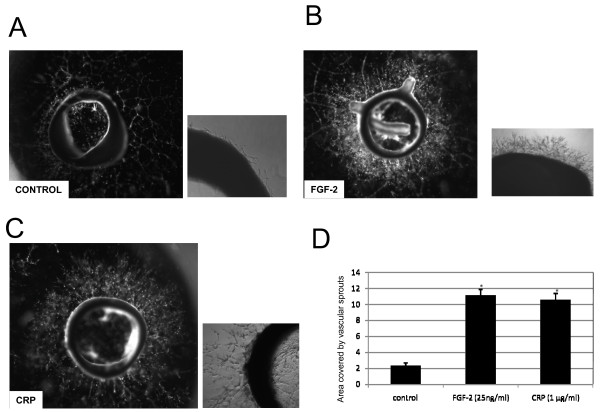
Aortic ring assay. A, control. B, Incubated with FGF-2 (25 ng/ml). C, Incubated with 1 μg/ml of detoxified CRP [CRPdt]. D, Bar graphs showing area covered by vascular sprouts in control, FGF-2 (25 ng/ml) and CRPdt (1 μg/ml).

We used a rat aortic ring assay to examine the effects of CRP on angiogenesis. We showed that CRP stimulated microvessel sprouting from the cultured aortic rings (area occupied by sprout microvessels of aortic rings; control, 2,3 ± 0,3; CRP (1 μg/ml) 10,5 ± 0,8; FGF-2 (25 ng/ml), 11,1 ± 0,03; p < 0.01). (Figure 7)

### CRP induces endothelial cell invasion in a 3D-Matrigel™ matrices

**Figure 8:**
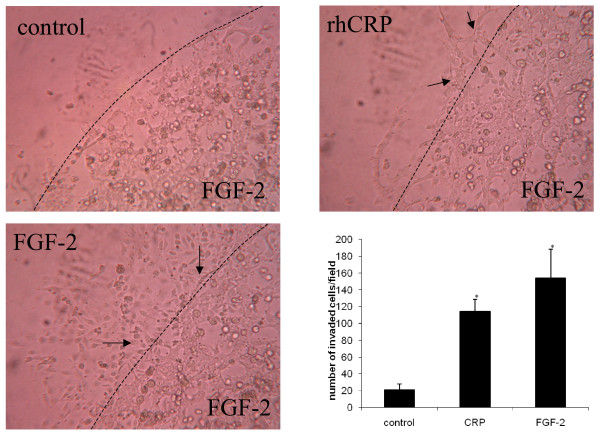
3D-matrigel invasion assay in control, FGF-2 (25 ng/ml)(arrows) and CRPdt (1 μg/ml)(arrows). Dotted line marks the interface between the first and the second layer of the matrigel.

We investigated the effects of CRPdt on invasion/migration using HCAECs in 3D-Matrigel™ matrices. Unstimulated HCAEC (control) showed a low level of network formation (Figure 8). However, when an HCAEC network was preformed in Matrigel™ in the presence of FGF-2 (25 ng/ml), CRPdt (1 ug/ml) stimulated an invasion into a second layer of 3D-Matrigel™ matrices (Figure 8, arrows).

### CRP increased the phosphorylation of ERK1/2

**Figure 9:**
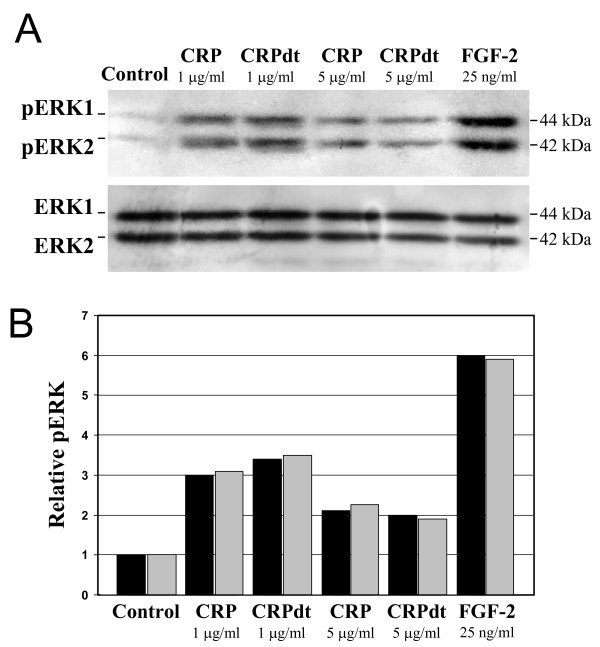
CRP activated ERK1/2 in HCAEC. A, Western blot showing the effect of 1–5 μg/ml CRP (non-detoxified and detoxified CRP [CRPdt]) on phosphorylation of ERK1/2 (pERK1/2) in HCAEC. The increase was similar to that produced by FGF-2 (25 ng/ml). Total ERK1/2 protein levels were used as a loading control. Experiments were performed at least twice and a representative example is shown. B, quantitative densitometic analysis of the blot shown in A. (ERK-1 [black bars]; ERK-2 [shaded bars]).

Phosphorylation of ERK1/2 is a key event associated with mitogenesis in vascular EC, and we have previously shown that its inhibition in BAEC is sufficient to prevent growth factor stimulation of angiogenesis [41]. Semi-confluent ECs (BAEC and HCAEC) cultured in SPM for 48 h were treated with CRP/CRPdt (1 or 5 μg/ml) or FGF-2 (25 ng/ml; positive control), and incubated for 8 min (optimum time for phosphorylation as determined in our previous published studies) [41]. Both CRP and CRPdt notably increased phosphorylation of ERK1/2 in HCAEC (Figure 9) and BAEC (data not shown). Neither purified endotoxin nor sodium azide induced ERK1/2 activation (data not shown).

### CRP stimulated gene expression of multiple angiogenic markers

HCAEC were treated for various times with CRP (see methods). Table 1 shows results obtained by quantitative TaqMan low-density array analysis corresponding to 6 genes involved in angiogenesis that increased more than 1.5 fold in CRP treated HCAEC compared to the control azide-only cells (1.0) in two separate experiments. These genes were two members of the Notch family (Notch1 and Notch3), VEGF receptor-2 (KDR), the main VEGF receptor, the mitogen platelet derived growth factor-beta (PDGFβ), cysteine-rich 61 (CYR61), which stimulates angiogenesis following integrin binding, and inhibitor of DNA binding 1 (ID1). No studied angiogenic genes were down-regulated compared with control cells.

**Table 1:** Angiogenesis-related genes modulated by CRP in HCAEC

Gene name	Fold-increase (time) Experiment #1	Fold-increase (time) Experiment #2	Gene function
KDR/Flk-1/VEGFR2	1.53 (12 h)	1.94 (24 h)	Cell survival, proliferation and angiogenesis^44^
PDGF-B	2.44 (24 h)	3.94 (12 h)	Proliferation and angiogenesis^45^
Notch1	3.28 (12 h)	1.97 (12 h)	Angiogenesis (spreading and branching)^46^
Notch3	2.03 (24 h)	4.46 (72 h)	Vascular cell identity and survival^47,48^
CYR61/CCN1	4.94 (72 h)	3.91 (12 h)	Connective tissue growth factor ^49^
ID1	2.65 (24 h)	1.94 (12 h)	Cell cycle and angiogenesis^50^

PDGF, Platelet-derived growth factor; KDR: kinase-insert domain receptor; VEGFR2, vascular endothelial cell growth factor receptor-2; CYR61, Cysteine-rich angiogenic inducer 6; ID1, Inhibitor of DNA binding/differentiation-1.

## Discussion

Several studies have suggested CRP as biomarker for cardiovascular and cerebrovascular diseases [13,14]; however, this protein also seems to be a mediator of atherosclerosis [26,27]. Angiogenesis is a recognised mechanism involved in the development of complicated atherosclerotic plaques and previous studies have provided controversial data regarding the possible angiogenic or anti-angiogenic effect of CRP. In this work, we have examined the hypothesis that CRP might play a role in modulating angiogenesis, and as such, its presence in vascular regions of developing arterial plaques may be implicated in their progression to unstable, haemorrhagic lesions prone to rupture. Our data shows that CRP, at concentrations commonly found in the circulation of patients with active carotid disease, is highly pro-angiogenic both *in vitro*, and *in vivo*. Furthermore, CRP activates cell signalling and increases expression of key genes associated with angiogenesis.

In order to show that the effects of CRP were due to CRP itself and not other components, we have attempted to address the controversy surrounding the influence of ¨contaminants¨ present in commercial preparations of CRP. Notably, Pepys *et al*. demonstrated that pure natural human CRP free from endotoxins and azide, was not pro-inflammatory to macrophages *in vitro *[42]. More specifically, inhibition of HUVEC cell proliferation, eNOS gene expression and increased apoptosis measured by activation of caspase-3/9, have been attributed to the presence of azide in CRP preparations. Similarly, contaminating LPS was shown to increase IL-8, ICAM-1 and MCP-1 gene expression [39,40]. It is worthy of note, however, that the effects of azide were demonstrated at concentrations (0.0025%) equal to those produced by administration of 50 μg/ml CRP. In our study, concentrations of CRP and therefore azide used in the majority of experiments were much lower and in fact, had no significant effects on cell growth and gene expression as demonstrated in the TaqMan microarrays. Even so, this highlights the importance of using satisfactory controls when conducting this type of assays. In our studies, all of the controls, used for comparison with CRP tests, contained equivalent amounts of azide so that direct comparisons were able to be made. Finally, in our study, we show that the treatment of CRP with detoxi-gel columns did not negate the pro-angiogenic effects of CRP. Interestingly, assays performed using purified LPS at concentrations that were similar to or exceeded those reported in high purity human recombinant CRP from our commercial supplier [43,44] demonstrated no significant effects on BAEC or HCAEC angiogenesis or IL-8 gene activation. This is in agreement with the recent findings of Dasu *et al*. who showed that purified CRP was able to activate IL-8, IL-6, IL-1β, PAI-1 and eNOS in Toll-like receptor 4 knockout HAEC, indicating that the effects were not due to LPS contamination [43].

In this study, we showed that CRP induces angiogenesis *in vitro *in two separate sources of primary cultured vascular EC as well as *in vivo *as judged by an increase in capillary formation in the CAM assay, aortic ring assay and 3D-Matrigel. The angiogenic potency of CRPdt was quite strong, being 50–75% of that demonstrated by our positive control FGF-2 (25 ng/ml; comparisons not included). One other study showed previously that CRP induced proliferation of rabbit thoracic EC with a concomitant increase in expression of p-ERK1/2, similar to our own findings [44]. In their study, they controlled for LPS by measuring levels with the Limulus assay which were recorded as < 0.125 EU/ml. The authors, however, found that increased proliferation occurred only at significantly high CRP concentrations of (> 20 μg/ml), and furthermore, they did not consider the effects of azide. In agreement with our findings, Bello et al (2008), showed that CRP increases VEGF-A expression via PI3-kinase and ERK1/2 pathway and thus could play a role in the angiogenesis process [45]. Orozlan *et al*. found no effects of CRP on HUVEC proliferation although they did not supply data indicating the concentration of CRP used [46]. In our previous studies where we have characterised various types of vascular cells, we have found HUVEC to be the least responsive to pro-angiogenic stimuli including VEGF, FGF-2 and oligosaccharides of hyaluronan; therefore, these results are perhaps not surprising [41]. The fact that CRP did not stimulate HCAEC proliferation suggests that there may be variability in cellular responses dependent on cell type, and EC have been shown in many studies to be heterogeneous in this regard. Indeed, in contrast to our results, one recent study showed induction of apoptosis in human umbilical EC (HUVEC) incubated with CRP, analyzed by TUNEL and caspase-3 activity assay and inhibition [47]. However, the above study used 10 μg/ml of CRP in their assay and we observed a pro-angiogenic effect of CRP at 1 and 5 μg/ml. Moreover, the apoptotic effect of CRP was demonstrated in HUVEC and cellular response may be dependent on EC origin. In addition, in the same study the authors showed that CRP treatment of monocluclear cells induced production of MMP-9 which is involved in extraclellular matrix degradation, cell migration and release of angiogenic factors necessary to elicit angiogesis [48,49] Verma *et al*. showed that purified CRP attenuated NO release in human saphenous vein EC, increased apoptosis and inhibited capillary-like tube formation in matrigel at 5–25 μg/ml [32]; however, they did not control for the effects of azide. The same authors also demonstrated that CRP inhibited EC progenitor differentiation, survival and function through a process involving a reduction in NO expression appeared to be carried out without the use of suitable azide and endotoxin controls [50].

Our data showing a potent angiogenic effect of CRP/CRPdt is strongly backed up by the results of our real-time TaqMan PCR microarrays. Using our specifically designed targeted microfluidity cards, we showed up-regulation of key genes involved in promotion of vascularisation. We found 6 genes up-regulated by CRPdt on HCAEC between 12–72 h. VEGF receptor-2 (KDR), the main receptor mediating both signal transduction and the biologic responses, including angiogenesis, triggered by VEGF in endothelial cells [51], was significantly induced by CRPdt. Up-regulation of this receptor together with growth factors such as PDGF- B involved in cell proliferation and angiogenesis [52], could be one mechanism through which CRP initiates its angiogenic effects. Notch1 was increased after 12 h of CRP treatment. Notch1 is required for formation of correct sprouting and branching patterns during VEGF-stimulated angiogenesis *in vivo *[53]. Recently, cyclic strain was shown to up-regulate Notch1 in human vascular EC and this process was responsible for significantly increased tube-formation in matrigel suggesting a role in development of atherosclerosis [54]. Notch3 is other member of the Notch family that seems to be critical for vascular cell survival and is required for the arterial identity and maturation of vascular cells [55,56]. One particularly novel finding was the up-regulation of CYR61. CYR61 is an extracellular matrix-associated protein expressed within developing vasculature, which promotes angiogenesis both in vitro and *in vivo *[57]. CYR61 binds directly to the integrin α_v_β_3 _present on activated EC and mediates chemotaxis and tube formation. Finally, ID1, originally identify as a dominant-negative antagonist of the basis helix-loop-helix (HLH) transcription factors has been recently involved in VEGF-induced angiogenesis in human endothelial cells [58]. Future work should be addressed to determine the expression, localization and relevance of these proteins in angiogenic regions of developing complicated atherosclerotic lesions.

This discussion would not be complete without a brief mention of the recently characterised modified CRP (mCRP). Evidence has emerged that native pentametric CRP can change its structural conformation following separation into monomers. Following re-arrangement, formation of the mCRP sub-unit has increased binding affinity for plasma membranes, and has been shown to be preferentially expressed in tissues [59]. Recent evidence suggest that mCRP may be a significantly weaker stimulator of pro-inflammatory molecules in vascular EC (e.g. IL-8, PAI-1 and prostaglandin F1-α), and hence atherogenic effects [60]. However, it is important to remember that native CRP can be found at high concentration in many hospital patients and most of them do not develop acute cardiovascular events. This suggests that cardiovascular effects of CRP if indeed important maybe due to its modified form.

## Conclusion

Our findings strongly suggest an important role for CRP in modulation of angiogenesis and as such, CRP could promote the formation of intimal neovessels of complicated unstable plaques increasing the likelihood of rupture. The distribution of both native and mCRP should be investigated in complicated atherosclerotic plaques and further studies should aim to identify the mechanisms of cell binding and intracellular mechanisms leading to cellular activation.

## Methods

### Materials

Human coronary artery EC (HCAEC), EC basal medium and growth factor supplements were bought from TCS CellWorks (Botolph Claydon, UK). HCAEC were used between passage 2 and 6, and cultured according to the manufacturer's instructions. Bovine aortic EC (BAEC) were isolated from bovine arteries, seeded in DMEM (Invitrogen) supplemented with 15% foetal calf serum, containing penicillin and 100 μg/ml streptomycin (Sigma-Aldrich, St Louis, MO) routinely cultured as described elsewhere, and used between passage 3 and 6.^41 ^Human recombinant CRP was obtained from Calbiochem (San Diego, California, USA). Sodium azide, phenylmethylsulfonyl fluoride (PMSF), leupeptin and lipopolysaccharide (LPS; 20,000 endotoxin units (EU)/mg LPS) were purchased from Sigma-Aldrich. Growth factor-reduced Matrigel was bought from Beckton Dickinson (BD Biosciences, San Jose, CA) and recombinant basic fibroblast growth factor (FGF-2) was from R&D systems (Minneapolis, MN). Antibody to CRP was purchased from Sigma. Experimental research carried on animals followed internationally recognized guidelines and was approved by local ethical committe at Centro de Investigación Cardiovascular, CSIC-ICCC, Hospital de la Santa Creu i Sant Pau, Barcelona, Spain.

### CRP purity testing and endotoxin removal

CRP purity was checked by SDS-PAGE followed by Coomassie blue staining where CRP was identified as a single band [57]. Endotoxin concentrations in CRP samples were measured using the Limulus assay (sensitivity < 0.125 EU/ml, Chromogenix AB, Mölndal, Sweden). Purified LPS was included in control experiments at concentrations that exceeded those reported to be found in human recombinant CRP from Calbiochem (1 ng/ml) [58,59]. In all of the experiments CRP treated with detoxi-gel columns (CRPdt) containing immobilized polymyxin B was used to ensure the absence of pyrogens (AffinityPak™ detoxi-Gel™ column; Pierce, Rockford, IL). Removal of LPS was confirmed using the limulus assay. Non-detoxified native CRP preparations were used for comparison. To analyze the possible effect of sodium azide present in the CRP preparations, cells were incubated with 0.00005–0.0005% sodium azide, equivalent to that found in 1–10 μg/ml CRP.

### Response testing of HCAEC and BAEC to CRP: choice of CRP concentration

In this work, we investigated the effects of CRP at concentrations ranging from 1–10 μg/ml. Published data suggests that normal circulating levels of CRP are < 1 μg/ml. In pathological situations, such as in patients with inflammation, and particularly in those with advanced unstable atherosclerosis, CRP levels increase. CRP levels of < 1, 1 to 3, and > 3 μg/ml correspond to low-, moderate-, and high-risk groups for future cardiovascular events [21].

### Chemotaxis assay

BAEC or HCAEC were seeded at 7.3 × 10^4 ^cells/ml in 100 μl of serum poor medium on Transwell porous membranes (Costar; 8-μm pore filter) plated into a 24-well plate. Basal medium supplemented with 0.1% FBS and CRP (1–5 μg/ml). We also incubed cells with CRP pre-treated with antibody to CRP in order to confirm the effect was due to CRP protein itself. FGF-2 (25 ng/ml) was used as a positive control. For each experimental condition, cells were treated in duplicate. After 24 h incubation, the cells which did not migrate on the upper surface of the membrane were removed with a cotton swab soaked with PBS then wiped with a dried cotton swab. The cells which had migrated were fixed with 4% paraformaldehyde, left to air dry, stained with Giemsa and counted with an optical microscope. All experiments were performed at least three times.

### Effect of CRP on BAEC and HCAEC proliferation

BAEC were seeded in complete medium at a concentration of 2 × 10^4^cells/ml (2 ml per well) in 6-well plates. HCAEC were seeded in complete medium at a concentration of 2 × 10^4 ^cells/ml per well in 24-well plates. After attachment (4 h) the medium was replaced with serum poor medium (SPM), containing 2.5% FBS (BAEC) and 5% FBS (HCAEC) in which the cells grew at a significantly reduced rate. CRP was added at concentrations ranging from 1–5 μg/ml. After 72 h incubation, cells were washed with PBS without Ca^2+ ^and Mg^2+^, detached with trypsin then counted using a Coulter counter (Coulter Electronics, Hialeah, FL). All experiments were performed at least three times.

### Tube-like structure formation assay

BAEC or HCAEC (2 × 10^6 ^cells/ml) were cultured in complete medium and mixed in an equal volume with 40 μl of growth factor-reduced Matrigel (10 mg/ml) with or without CRP at concentrations varying between 1 and 5 μg/ml. We also incubed cells with CRP pre-treated with antibody to CRP in order to confirm the effect was due to CRP protein itself. FGF-2 (25 ng/ml) was used as a positive control. Half of the mixture was allowed to polymerise as three-dimensional droplets in 48-well plates and each experimental condition was carried out in duplicate. After polymerization (1 h), each spot of Matrigel was bathed in 500 μl of complete medium for 24 h. The cells were then fixed with 4% paraformaldehyde for 10 min. the total length of tube-like structures was measured by light microscopy after 48 h in a double-blinded fashion. All experiments were performed at least three times.

### Chick Chorioallantoic membrane (CAM) assay

The angiogenic activity of compounds was determined in the chick chorioallantoic assay (CAM) as described previously [61]. CRPdt (5 μg) was applied to the membrane (10 membranes per treatment) and the resultant angiogenesis scored as 0, negative; 0.5, change in vessel architecture; 1, partial spoke wheel (1/3 of circumference exhibits directional angiogenesis); 2, spoke wheel; 3 or greater, strong and full spoke wheel. Control membranes had the equivalent concentration of sodium azide added. To photograph the membrane, 2 cm^3 ^of a 50% emulsion of aqueous paraffin oil containing 2% Tween-80 was injected at the site of application and photographed using a Leitz dissecting microscope. FGF-2 (100 ng) was used as a positive control. All experiments were performed at least three times. This approach enabled calculation of an accumulated response in each group. All experiments were performed at least five times and statistical differences were determined by the Mann-Whitney U test and the data is expressed as a median value (m).

### Rat aortic ring sprouting assay

Aortic ring assays were performed as described with minor modifications [62]. Aortas were removed from adult male Wistar rats (250 g) and immediately placed in ice cold EC growth medium (ECGM) supplemeted with 1% PBS. After removing the fibro-adipose tissue, arteries were cut into 1 mm long cross section, rinsed in PBS and placed on the Matrigel-coated wells. Artery rings were covered with addition layer of Matrigel (10 mg/ml) and incubated with ECGM at 37°C in 5% CO_2_. After 24 hours incubation, medium was replaced with medium ECGM with or without CRP (1 μg/ml). FGF-2 (25 ng/ml) was used as a positive. After 6 days, images of aortic rings were taken using a Leica DMIRE microscope and the outgrowth area delineated and measured. Sprouts was measuring using Image software and analyzed by calculating the area occupied by microvessel sprouts eminating from the arterial ring.

### Invasion assay in a 3D-Matrigel™ matrix

To assess the effect of CRP on EC invasion into a second Matrigel™ layer the first layer was formed in 0.1% FCS and 25 ng/ml of FGF-2 and tube-like structures allowed to form over 24 h. Then a second layer of Matrigel™ was formed around the first layer containing 1 μg/ml of CRP. After polymerisation, these wells were incubated with 500 μl of medium containing 0.1% FCS with or without 1 μg/ml CRP or with 25 ng/ml FGF-2 as a positive control for a further 24 h. The wells were fixed with 4% PFA and the number of invading cells was quantified by visualising the border with the Matrigel™ using phase contrast microscopy and counting the number of cells in three different areas of the second matrix layer. Experiments were performed in triplicate wells.

### RNA extraction, cDNA synthesis and TaqMan Low-Density Array

Our pilot studies demonstrated up-regulation of IL-8, (used as a marker of EC activation) by CRP [60], at concentrations ranging from 1–25 μg/ml. ECs (BAEC and HCAEC) we incubated for 4–72 h in presence or absence of CRP and IL-8 expression was assessed by real-Time PCR. IL-8 expression was not abrogated by pre-incubation with polymyxin B (5 μg/ml) used to exclude the potential effect of traces of LPS potentially present in CRP preparations. RNA was isolated from semi-confluent cultured HCAEC in SPM following treatment for 4–72 h with CRP (5 μg/ml) or sodium azide alone using the RNeasy™ Mini Kit (Qiagen) according to the manufacturer's instructions. The TaqMan low-density array (Applied Biosystems) consists of 48 selected TaqMan primers and probes (see Additional file 1) preconfigured in a 384-well format and spotted on a microfluidic card (2 replicates per assay). Control HCAEC and those treated with CRP for different times (4 h, 12 h, 24 h and 72 h) were prepared in triplicate, then the three wells were pooled together. 50 μl of cDNA from each sample was loaded on the TaqMan Low-Density Array as described [5]. Real-time RT-PCR amplifications were run on an ABI Prism^® ^7900 Ht sequence Detection System (Applied Biosystems) with a TaqMan Low-Density Array Upgrade. Thermal cycling conditions were as follows: 2 min at 50°C; 10 min at 95°C; 40 cycles of denaturation at 95°C for 15 seconds; and annealing and extension at 60°C for 1 min. The experiment was performed twice and only genes up-regulated in both experiments were considered validated.

### Western blotting

BAEC or HCAEC were seeded in complete medium in 24-well plates. After 48 h incubation, the medium was replaced with SPM for 48 h before addition of CRP (1–5 μg/ml) or 25 ng/ml FGF-2 for 8 min and western blotting was carried out using standard process as previously described [41]. Proteins were electroblotted (Hoefer, Bucks, UK) onto nitrocellulose filters (1 h) and the filters were blocked for 1 h at room temperature in TBS-Tween (pH 7.4) containing 1% BSA. Filters were stained with the following primary antibodies diluted in the appropriate blocking buffer, overnight at 4°C on a rotating shaker: rabbit monoclonal antibodies to ERK1/2 (1:1000) and mouse monoclonal antibodies to phosphorylated ERK1/2 (P-ERK1/2, 1:1000) from Santa Cruz Biotechnology. After washing filters were stained with either goat anti-rabbit or rabbit anti-mouse horse-radish peroxidase-conjugated secondary antibodies (1:1000, 1 h, room temperature). Proteins were visualized using ECL chemiluminescent detection. All experiments were performed at least twice.

## Authors' contributions

MMT carried out RNA extraction, cDNA synthesis and TaqMan Low-Density Arrays. SM and  MGO performed all cell culture studies, migration and proliferation assays. AL performed chemotaxis assays. DW carried out Chick Chorioallantoic membrane (CAM) assays. CR carried out pilot studies on the effect of CRP on IL-8. JK analysed all the results. MS, LB, JMG and JK participated in the design of the study and preparation of the manuscript. All authors read and approved the manuscript.

## Supplementary Material

Additional file 1 Applied bio systems codes. Click here for file
