# Supplementary material for: C-reactive protein exerts angiogenic effects on vascular endothelial cells and modulates associated signalling pathways and gene expression
Source: BMC Cell Biol. 2008 Sep 2;9:47. doi: 10.1186/1471-2121-9-47 (PMC2551596; doi:10.1186/1471-2121-9-47)
Supplement: Additional file 1 — Applied bio systems codes. [file 1471-2121-9-47-S1.doc]

| **Gene name** | **Gene name** | **applied biosystems code** |
| --- | --- | --- |
| Tyrosine kinase endothelial | TEK | Hs00176096_m1 |
| Platelet-derived growth factor beta | PDGFB | Hs00234042_m1 |
| Vascular endothelial growth factor | VEGF | Hs00173626_m1 |
| Matrix metallopeptidase 9 | MMP9 | Hs00234579_m1 |
| Neuropilin 1 | NRP1 | Hs00818574_m1 |
| Integrin alpha V | ITGAV | Hs00233808_m1 |
| Interlekin 6 | IL6 | Hs00174131_m1 |
| Matrix metallopeptidase 1 | MMP1 | Hs00233958_m1 |
| C-X-C motif ligand 12 | CXCL12 | Hs00171022_m1 |
| Trombospondin 1 | TMSB4Y | Hs00795219_m1 |
| Vascular endothelial growth factor receptor 2 | KDR | Hs00176676_m1 |
| Thrombospondin 1 | THBS1 | Hs00170236_m1 |
| Transforming growth factor beta 1 | TGFB1 | Hs99999918_m1 |
| Matrix metallopeptidase 8 | MMP8 | Hs00233972_m1 |
| Notch homolog Drosophila 2 | NOTCH2 | Hs00225747_m1 |
| Neuronal cell adhesion molecule | NRCAM | Hs00170554_m1 |
| Wingless-type MMTV integration site family member 2B | WNT2B | Hs00244632_m1 |
| Platelet-derived growth factor receptor beta | PDGFRB | Hs00182163_m1 |
| Inhibitor of DNA binding/differentiation-1 | ID1 | Hs00357821_g1 |
| Integrin beta 3 | ITGB3 | Hs00173978_m1 |
| Ribosomal | 18S | Hs99999901_s1 |
| Epidermal growth factor | EGF | Hs00153181_m1 |
| colony stimulating granulocyte factor 3 | CSF3 | Hs00738431_g1 |
| Vascular endothelial growth factor receptor | VEGFB | Hs00173634_m1 |
| tyrosine kinase with immunoglobulin-EGF-like domains1 | TIE1 | Hs00178500_m1 |
| Angiopoietin 1 | ANGPT1 | Hs00181613_m1 |
| Signal transducer and activator of transcription q | STAT1 | Hs00234829_m1 |
| Laminin gamma 1 | LAMC1 | Hs00267056_m1 |
| Endoglin (cd105) | ENG | Hs00164438_m1 |
| Integrin Beta 1 | ITGB1 | Hs00236976_m1 |
| Cystein-rich angiogenic inducer | CYR61 | Hs00155479_m1 |
| Placental growth factor | PGF | Hs00182176_m1 |
| Notch homolog Drosophila 1 | NOTCH1 | Hs00413187_m1 |
| Vascular endothelial growth factor receptor 1 | FLT1 | Hs00176573_m1 |
| AP1 gamma subunit binding protein 1 | AP1GBP1 | Hs00373054_m1 |
| Notch homolog Drosophila 3 | NOTCH3 | Hs00166432_m1 |
| Beta Glucuronidase | GUSB | Hs99999908_m1 |
| Wingless | WNT1 | Hs00180529_m1 |
| Osteonectine | SPARC | Hs00277762_m1 |
| Heparin-binding EGF-like growth factor | HBEGF | Hs00181813_m1 |
| Activating transcrition factor 3 | ATF3 | Hs00231069_m1 |
| Hypoxia inducible factor alpha 3 | HIF3A | Hs00541709_m1 |
| Integrin beta 1 binding protein 2 (melusin 2) | ITGB1BP2 | Hs00183746_m1 |
| Prostaglandin sintasa 2 | PTGS2 | Hs00153133_m1 |
| Notch homolog Drosophila 4 | NOTCH4 | Hs00270200_m1 |
| Alanyl aminopeptidase | ANPEP | Hs00174265_m1 |
| Matrix metallopeptidase 2 | MMP2 | Hs00234422_m1 |
| Matrix metallopeptidase 3 | MMP3 | Hs00233962_m1 |
